# Horizon scanning process to foresight emerging issues in Arabsphere's water vision

**DOI:** 10.1038/s41598-022-16803-1

**Published:** 2022-07-26

**Authors:** Ayman Batisha

**Affiliations:** 1 Environment and Climate Change Research Institute, National Water Research Center, Cairo, Egypt; 2 Council of Future Studies and Risk Management, Academy of Scientific Research and Technology (ASRT), Ministry of Scientific Research, Cairo, Egypt

**Keywords:** Environmental sciences, Hydrology, Engineering

## Abstract

The Arabsphere struggles with highly complicated water challenges due to climate change, desertification, coronavirus pandemic, and Russo-Ukrainian War. This paper explores how to build a robust water vision to pave the road to achieving sustainable development goals (SDGs) in the Arabsphere. A sustainable water future (SWF) necessitates an interdisciplinary and transdisciplinary research strategy. ‘Horizon scanning’ process (HSP) is one of the promising foresight methodologies. A generic process for “Horizon scanning” has been developed to cope with water crises and challenges. “DEEPEST” holistic framework has been designed to suit both the “Futurology” science and water, environment, and engineering disciplines. “DEEPEST” characterizes Demographics, Ecological, Environmental, Political, Economic, Social, and Technological features. The macro-future factors (MFF) applied in the foresight process (FP) have been presented. The results showed that Water conservation (WC), Circular Water (CW), and Emerging Water Technologies (EWTs) were the main outcomes of the ‘Horizon scanning’ process (HSP). The paper concluded that the preparing for a sustainable water future (SWF) must be right now and the opportunities range from the deepest water drop to the highest water drop on Earth. The essence of the conclusion is hydrosphere sustainability, particularly in Arabsphere, should be given extreme concentration, effort, and support.

## Introduction

Water is equal to life. Freshwater resources mainly mean drinking, cooking, and hygiene. Water resources represent vital input for many human activities and industries, examples involve the construction industry and many other industries. The state of the Arab fresh water resources (WRs) reflects the hyper aridity of the Arab climate. The Arabsphere contains 5% of the world’s population with only 1% of its freshwater resources. The majority of the twenty-two Arab countries suffer from water scarcity. The Arabsphere’s climate is getting warmer, drier and more variable. Millions of people could be displaced. Climate change causes uncertainty to water related decisions, increase temperatures, shorten growing seasons and is likely to change precipitation patterns. More intensive droughts may lead to a reduction in crop production and put more stress on available water resources. Sea level rise could affect many cities and large regions of the most populated zones, especially, in Arabic rivers deltas.

Nowadays, Arab countries are also exposed to coronavirus pandemic which has negative impacts on Arab people’s well-being. Various forecasting and prediction models for COVID-19 have been applied to make appropriate control measures. So, only some examples may be cited. The coronavirus disease 2019 outbreak in China, Italy and France has been analyzed^1^. Forecasting of COVID-19 spread in Brazil, India, Peru, Russia and the USA has been modeled^2^. Forecasting the death rate of COVID-19 in the world using time series models has been modeled^3^.

There is a need for systematic categorization to facilitate recognition and wise preparation for possible future drivers to achieving sustainable development goals (SDGs) in the Arabsphere. The prime objective is to build a robust water vision to pave the road by enabling a SWF. Foresight science and discipline could be considered a protective tool in guiding future-oriented strategies. Foresight studies are used in designing science, technology, and innovation (STI) policies. This work investigated one of the most promising foresight techniques to be utilized to foresee and guide a complicated and cross-disciplinary Arabic water vision. Three Horizons is a method developed to be applied in future studies and has much strength when utilized for “emerging issues” applications^4^. As a pathway for transformation, Three Horizons could be applied to uncertain futures and complex problems. The characteristics and opportunities of “Three Horizons” have been discussed. “Three Horizons” could explore how Global environmental change, climate change, and other transformations could be achieved^5^.

Future technological impact and challenges have been provided relating to (STI) policies for the Future^6^. The techniques utilized for HS and scan horizons have been outlined with special emphasis on Trends, Drivers, Weak Signals, Wild Cards, and Discontinuities. Significant tools for scan horizons and HS have been presented and focused in terms of Reviewing, Brainstorming, Surveys, Network Analysis, Big Data, Bibliometrics, and Semantic Analysis^7^. “HS” has been implemented to foresee the radical innovation breakthroughs as preparation for policy intelligence and implementation of Horizon Europe’s research and innovation programs^8^. An example that represents the Russian experience in Foresight has been described^9^. HS could be an approach for exploring new S&T opportunities^10^. The example has focused on Russian Foresight in the science and technology (S&T) field which could be useful for other states with similar features, priorities, constraints, and barriers. Foresight activities and how to be designed for Creative Futures and future repositioning focused have been reported^11^.

Nature-based solutions focuses on sustainable pathways, green growth, sustainable urbanization, infrastructure (blue and green) and ecosystem (mitigation and adaptation), resilience and future societies^12^. Nature-based solutions could be a pathway to circular economy (CE), strengthen equitable future, support water security, establish maximum advantage for green and grey infrastructures (investments) and achieve sustainable water development for all^13^. As a pathway to sustainable development (SD), circular economy (CE) could support sustainable, equitable efficient and reasonable resources utilization^14^.

## Methods

### Study area

The Arabsphere includes any member country of the Arab League. The Arabsphere has four sub-regions (Arab Gulf, Horn of Africa, Maghreb (western Arabsphere), and Mashreq (eastern Arabsphere). Arab Gulf States include Bahrain, Emirates, Kuwait, Oman, Qatar, and Saudi Arabia. Maghreb States include: Morocco, Algeria, Tunisia, and Libya. Mashreq States include: Egypt, Palestine, Lebanon, Jordan, Syrian, and Iraq. The least developed countries (LDCs) include: Mauritania, Sudan, Somalia, Djibouti, the Comoros and Yemen.

### Sustainable development goal on water and sanitation (SDG 6)

SDG 6 is focusing on the sustainable water resources, used water and ecosystems. There are many global indicators to assess progress towards the targets of SDG 6. The global indicators are drinking water, sanitation, hygiene, used water, water quality, water-use efficiency, water stress, Integrated Water Resources Management (IWRM), transboundary, ecosystems (water-related ecosystems), cooperation and participation. There are four key components of IWRM implementation: (I) Enabling Environment, (II) Institutions and Participation section, (III) Management Instruments, (IV) and Financing. The latest year of reporting is 2017 for Country (or area), regional and world data. Based on UNEP data source, and defined by a score (0–100), [Units = %] the Arabsphere status of IWRM has been indicated. Degree of IWRM implementation and Final water resources management (WRM) Score are shown in Table 1 for Arabsphere, and for geographical regions and the whole world are shown in Table 2. Among the 19 IWRM reporting Arab countries, 63% are improbable to meet the global IWRM target unless progress is considerably improved^15^.

**Table 1:** Degree of IWRM achievement in the Arabsphere (0–100).

	Country	Region	I*	II*	III*	IV*	WRM
1	Algeria	Maghreb	40	42	51	60	48
2	Bahrain	Arab Gulf	28	48	43	40	40
3	Comoros	LDCs	27	35	14	28	26
4	Djibouti	LDCs					0
5	Egypt	Mashreq	47	42	49	24	40
6	Iraq	Mashreq	24	22	42	12	25
7	Jordan	Mashreq	68	57	70	58	63
8	Kuwait	Arab Gulf	84	82	80	80	82
9	Lebanon	Mashreq	37	40	40	12	32
10	Libya	Maghreb	57	45	53	32	47
11	Mauritania	LDCs	53	51	33	44	45
12	Morocco	Maghreb	68	69	64	55	64
13	Oman	Arab Gulf	33	18	57	24	33
14	Qatar	Arab Gulf	55	100	89	85	82
15	Saudi Arabia	Arab Gulf	42	68	71	46	57
16	Somalia	LDCs	13	13	11	4	10
17	State of Palestine	Mashreq					0
18	Sudan	LDCs	37	44	44	34	40
19	Syria	Mashreq					0
20	Tunisia	Maghreb	67	53	58	40	55
21	United Arab Emirates	Arab Gulf	59	90	71	80	75
22	Yemen	LDCs	50	51	36	20	39

**Table 2:** Degree of IWRM achievement in the world and main regions (0–100).

	Country	I*	II*	III*	IV*	WRM
1	Australia and New Zealand	75	67	74	72	72
2	Central and Southern Asia	40	42	38	30	37
3	Eastern and South-Eastern Asia	53	55	55	48	53
4	Europe and Northern America	70	70	70	59	67
5	Latin America and the Caribbean	33	39	40	27	35
6	Northern Africa and Western Asia	54	58	59	49	55
7	Sub-Saharan Africa	45	47	38	32	40
8	World	51	53	51	41	49

### Water use efficiency

Water-use efficiency (WUE) can be defined as the economic value added per volume of water withdrawn, by an activity. Examples of economic activities include agriculture, fishing, forestry, mining, quarrying, manufacturing, constructions and energy. Agriculture, industry, municipal water and energy sectors have high water use. According to^16^, WUE is an indicator defined to measure the value added per water unit, for an economic sector over a time, and expressed in USD/m^3^. Based on^16^, WUE is shown in Table 3 for the Arabsphere.

**Table 3:** WUE in the Arabsphere in USD/m^3^.

	Country	Region	WUE
1	Algeria	Maghreb	15.5
2	Bahrain	Arab Gulf	45.1
3	Comoros	LDCs	20.4
4	Djibouti	LDCs	–
5	Egypt	Mashreq	3.8
6	Iraq	Mashreq	1.3
7	Jordan	Mashreq	26.5
8	Kuwait	Arab Gulf	70.7
9	Lebanon	Mashreq	23.3
10	Libya	Maghreb	18.5
11	Mauritania	LDCs	1.9
12	Morocco	Maghreb	7.1
13	Oman	Arab Gulf	32.3
14	Qatar	Mashreq	233.9
15	Saudi Arabia	Arab Gulf	19.4
16	Somalia	Arab Gulf	0.1
17	State of Palestine	LDCs	15.7
18	Sudan	LDCs	1.6
19	Syria	Mashreq	2.8
20	Tunisia	Maghreb	10.8
21	United Arab Emirates	Arab Gulf	69.8
22	Yemen	LDCs	7.3

The main economic sectors (ES) related to this concept are agriculture (A), industry (I) and services (S). WUE is the sum of water-use efficiency of economic sectors [(*A*_WUE_), (*I*_WUE_), (*S*_WUE_)], weighted to the ratio of water used by each sector over the total uses (*A*_r_, *I*_r_, *S*_r_), using the formula:

$${\text{WUE}} = A_{{{\text{WUE}}}} \times A_{{\text{r}}} + I_{{{\text{WUE}}}} \times I_{{\text{r}}} + S_{{{\text{WUE}}}} \times S_{{\text{r}}}$$

$${\text{Knowing that}},{\text{ Water Efficiency for an economic sector }}\left( {ES_{{{\text{WUE}}}} } \right)\, = \,ES_{{{\text{WUE}}}} \, \times \,ES_{{\text{r}}}$$

where ES_WUE_ is the WUE for the economic sector (ES) index, ES_r_ is the ratio (weight) of water utilized by an economic sector (ES) over the total water.

WUE is a key water indicator in the set of SDGs^17^. WUE is the core of SDG goal 6, target 6.4, and indicator 6.4.1. It is hoped that WUE will be increased by 2030, as a promising step to mitigate water scarcity, especially, in water-scarce countries such as Arab countries. WUE could provide an analytical instrument for assessing the uncertainty and future water trends in changing environments, climates, and socioeconomic. For Time Interval of First Horizon, Water Withdrawals (Agriculture, Industries, and Municipalities) and Incremental evaporation, from irrigation, over wetlands, over open water, and Reservoir evaporation, could be assessed. To proceed towards other Time Intervals, corresponding to both Second Horizon and Third Horizon, all Demographics, Ecological, Environmental, Political, Economic, Social, and Technological (DEEPEST) dimensions should be investigated.

From WRs discipline perspective, normally rigorous scientific bases should be followed, then specialized knowledge could be applied. The rigorous scientific rule could be “the total quantity of Water Resources should be greater than (or equal) the total quantity of consumed Waters, in the same time period”. In each country or geographic region, Water Resources, Users, and consumers should be clearly identified within the specified time frame. As an example, although Desalination could be a sustainable water resource in many geographic regions, it is not a feasible water resource in Landlocked Countries.

### DEEPEST holistic framework feasible and future water resources

A sustainable water future (SWF) necessitates an interdisciplinary, transdisciplinary, and multidimensional vision. SWF environs Demographics, Ecological, Environmental, Political, Economic, Social, and Technological (DEEPEST) dimensions. The study attempted to simplify the methodology as much as possible.

**Figure 1:**
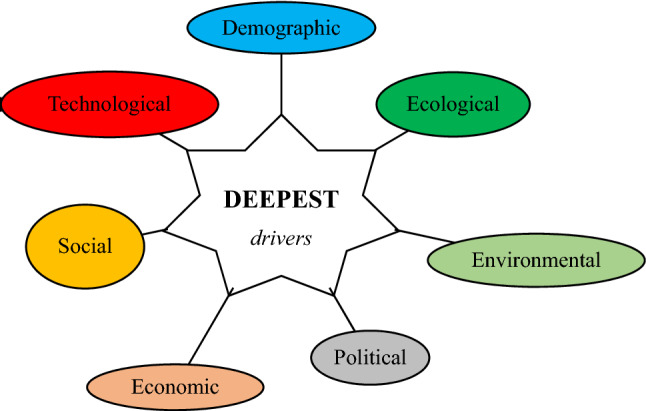
DEEPEST holistic framework domains.

In this study, a holistic framework is suggested to be wide applicable, especially in Hydrosphere discipline. An acronym "DEEPEST" has been suggested to refer to Demographic, Ecological, Environmental, Political, Economic, Social, and Technological drivers (or domains). This study defines DEEPEST holistic framework as a foresight tool for deepening the future vision, deciding dimensions of *future* assessment, foreseeing innovative *future* solutions, and enhancing *future developments.* DEEPEST holistic framework domains are displayed in Fig. 1. The purpose of Fig. 1 is to shed the light on "DEEPEST" road map domains. The key idea is that a Sustainable water future (SWF) could be considered as a function of Demographic, Ecological, Environmental, Political, Economic, Social, and Technological (DEEPEST) dimensions. "DEEPEST" holistic framework could be considered as a modified version of PESTEL, PESTLE, SLEPT, STEPE, STEEPLE, STEEPLED, PESTLEE, DESTEP, SPELIT, PMESII-PT, STEER, and TELOS frameworks. "DEEPEST' holistic framework has been designed to suit the “Futurology” science and discipline.

DEEPEST holistic framework may be considered as a modification for PEST (political, economic, socio-cultural and technological) analysis to adapt with freshwater challenges and hydrosphere sustainability. Recently, many researchers have used different forms of PEST assessment to analyze academic and practical applications. The Coronavirus Pandemic and higher education developments has been examined based on PEST methodology^18^. Sustainable management of healthcare waste due to COVID-19 outbreak has been identified based on PESTEL (political, economic, socio-cultural, technological, legal and environmental factors) assessment^19^. A future transport by means of suborbital flight has been studied using PESTLE (political, economic, social, technological, legal and environmental) analysis^20^. The urban sustainability has been assessed using STEEP -or PESTE- (social, technological, economic, environmental and political) framework^21^.

### Horizon scanning process (HSP)

In this century, the HSP has been highly developed, in particular, stakeholder analysis, interrelationships, interactions, impacts, and crossing effects investigation. The methodology that has been chosen for this research “HS” is one of the well-accepted Foresight methodologies. “Foresight” could be considered as the ability to foresee the future wisely. HS is mainly about identifying possible futures, possible signals, and trends. The verb “foresee” has been used rather than the verb “predict”. The verb “foresee” is a transitive verb that means to see (project, development, potential, consequences … etc.) beforehand, and the noun “Foresight” means to act, process, or result of “foreseeing” or “foresighted”.

Foresight techniques are used mainly for strategic planning, developing visioning, and optimizing future priorities. HSP is an emerging framework that could be utilized in the Foresight process. HS is a technique to foresee about future, anticipate probable and possible future visions and explore how the future could be designed and shaped. Some useful definitions for “HS” have been suggested. Definition introduced by^22^ explains that “horizon” scanning implies it goes beyond probable or even plausible into the whole scope of possible futures. Another feature is considering that “Horizon Scanning” is equivalent to “Environmental Scanning”, the main concern is to explore opportunities, change, challenges, and future developments. Another perspective for “Horizon Scanning” is introduced by^23^ where the concentration is on the change and its early warning signs. The idea of developments and detecting its early signs has been investigated^24^. The perspective of “Horizon Scanning” introduced by^25^ is focusing on the idea of foresight and the thinking in the range of medium and strategic future. Some definitions of horizon scanning, the concentration is on the policy-making process (emerging issues, uncertainties, opportunities, risks, threats, decision making, mitigation and exploitation) have been provided^26^.

**Figure 2:**
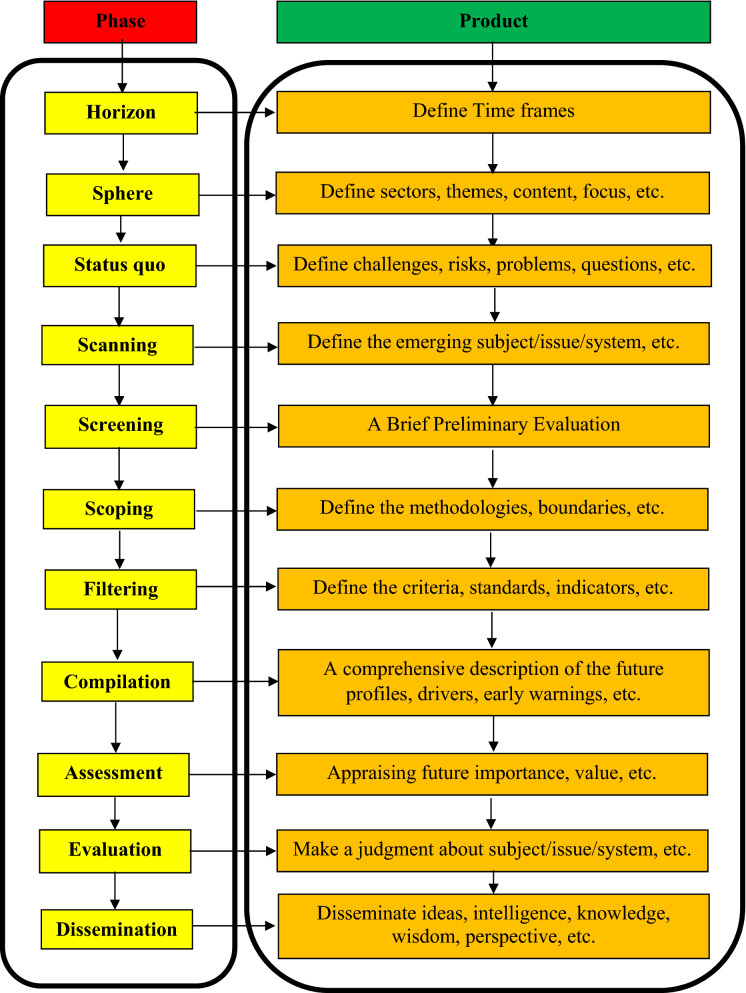
Flow diagram of the HS generic process.

Several *horizon scanning (HS)* procedures have been applied by International organizations^23–26^, and some academic scholars [^27–31^]. Flow diagram of the HS generic process is displayed in Fig. 2. The flow diagram in Fig. 2 facilitates the idea of the Horizon scanning process for audiences from the non-futurology disciplines such as water resources, environment, and engineering disciplines. Main functions of HSP are exploring innovative ideas, and patterns of future changes. Comparable to the key concepts in geometry, (Points, Lines, Curves, Planes, and Surfaces), the important axioms in HSP are *Signals,* (data points), *Trends* (Lines or Curves), *Drivers,* Emerging issues (EIs), and *Uncertainties* (Planes and Surfaces). Main concerns of HSP are weak signals, misleading signals, unconscious biases due to narrow vision, high-impact low-probability events, predictable events, and not-expected extreme events.

**Figure 3:**
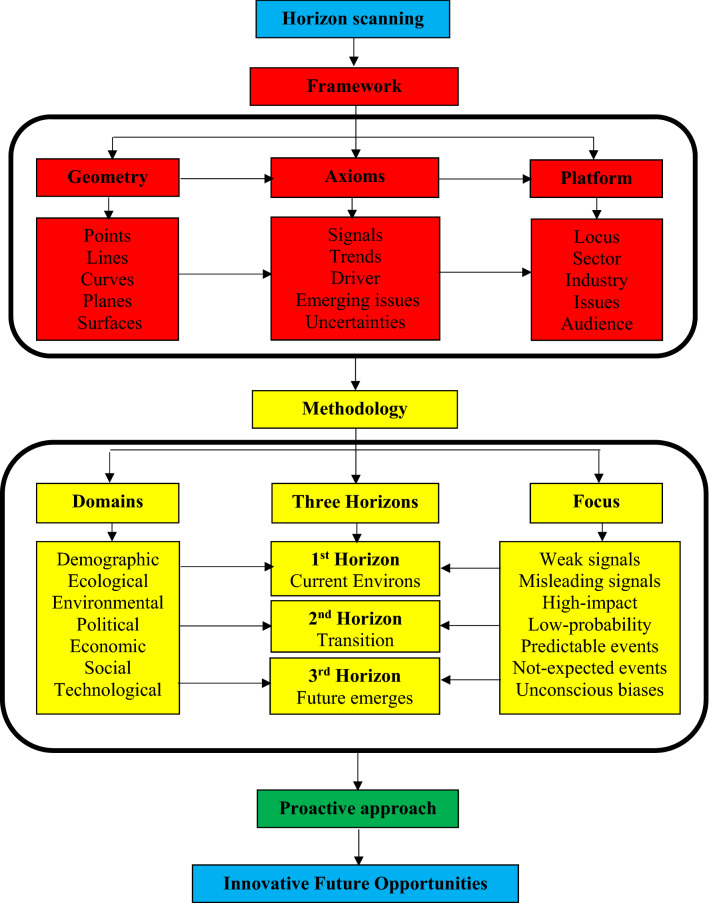
Horizon scanning process: engineering perspective.

The practical application has been implemented based on *Three Horizons* methodology. The first Horizon (H1) represents the current environs as if remains in future. The second Horizon (H2) is a transition interval in which innovative future opportunities are generated. The third Horizon (H3) is when idealistic and innovative ideas concerning the future emerge. Three Horizons methodology implies a dynamic situation composed of three paths move forward in time to future. These paths can harmonize complexity of the emerging issues (EIs) by impacting each other. The concentration of foresight process is the mid-term (H2) to long-term (H3). Engineering Perspective of HSP is displayed in Fig. 3.

The HSP has been initiated by desk research and reviewing the literature and outputs of available sources. Surveying publications of experts, specialists, and stakeholders, for collecting, Clustering, and Listing ideas has been a fundamental stage. Then, generating ideas and motivating creativity for HS based on a “DEEPEST” framework. Wild ideas have been generated by scanning new perspectives and assumptions. Then, a Matrix has been created to identify Themes and Emerging Water Technologies for the purpose of formulating the HSP. The activities have been tailored to suit the contents of the WRs discipline. The new reality could prove that COVID-19 lockdown, the Russo-Ukrainian war, and Grand Ethiopian Renaissance Dam (GERD) (construction and failure) and their related impact on the Water-Food-Energy-Nexus are good new examples of wild cards in the context of sustainable water future (SWF) in the Arabsphere.

Barriers and limitations have included inadequate resources, absence of stakeholders, misconception, uncertainty, fuzzy and cognitive and cultural barriers, forecast-based dominant culture, future illiteracy, cognitive biases, and difficulty to assess the impact or benefits of HS. The most important limitation is the uncertainty and fuzzy nature of HS.

## Results

The DEEPEST holistic framework is a process of systematic categorization to facilitate recognition and intelligence of possible future drivers for specific context. DEEPEST holistic framework establishes an integrated framework of macro-future factors applied in the HS component of foresight process. DEEPEST holistic framework of macro-future factors applied in the FP is presented in Table 4.

**Table 4:** DEEPEST holistic framework of macro-future factors.

Drivers	Key factors
Demographic	Population, Human demographics, Population growth, Overpopulation, Population densities, Age distribution, Religious Practice, Culture, Tradition, Gender, Fertility, Reproductive health, Mobility, Disabilities, Migration, Replacement migration, Refugees, Mortality, Animal demographics, Employment Status, Urbanization, Living Standards, Home Ownership, and Income Level
Ecological	Ecological features, Nature conservation, Human ecology, Biogeochemical cycle, Biodiversity, Life process, Living organisms, Diversity, Ecosystems, Community, Population, Species, Individuals, Adaptation, Biotic components, Abiotic components, Ecosystem processes, Terrestrial ecosystems, Ecosystem functions, Ecosystem services, Ecosystem resilience, Sustainable ecosystem, Carrying capacity, Biogeochemical processes, Nutrient cycles, Natural processes, Natural ecosystems, Semi-natural ecosystems, Forests ecosystems, Cultivated systems, Agroecosystems, Biotechnologies, Land use, Landscape, Wetlands, Urban areas, Restoration, Recreation, Aquatic ecosystems, Ecohydrology, Hydrological processes, Water resources, Fisheries, Marine ecosystems, Freshwater ecosystems, and Pollutant cycles
Environmental	*Water* (hydrological cycle, natural inflow, actual inflow, renewable water resources, rainfall, surface water, groundwater, evaporation, transpiration, evapotranspiration). Aquatic environment (sea level rise, water surface, etc.), Hydrogeochemistry, and Drought. Water quality and security, Drinking water, irrigation system, salinity, and drainage system. Used water treatment, and Emerging contaminants Global change, Natural (physical, chemical, and biological) cycles, Global warming, Solar energy, Renewable energy, *Climate change*, Climate variations (precipitation, temperature, humidity, wind), Greenhouse gas inventory, Carbon emissions, adaptation and mitigation strategies Geological conditions, Topography, Land environment, Land surface temperature (LST), Low-lying delta areas, **Soil** (composition, profile, texture, structure, moisture, saturation, field capacity, infiltration rate, erosion, salinization, salinity, and sodicity) *Sustainable Development*, Desertification, Human–environment interactions, Deforestation, Vulnerable eco-systems, Natural disasters, Ecological disasters, Biotoxins, Parasites prevalence, Wood supply, Invasive alien species, Environment issues, Genetically modified organisms, Food Modification, Nanomaterials, Microplastics, Pollution, Environmental Infringement, Environmental risk maps, and Environmental risk management
Political	Global forces, Global Cooperation, Global solidarity, Geopolitical, Policy cycle, Policy frameworks, Political stability, Political conflicts, Distributive policies, Regulatory policies, Constituent policies, Redistributive policies, Biofuels policy, Civil unrest, Peace, Conflict, War, Health policy, Education policy, Environmental policy, Water policy, Tax policy, Trade policy, The Agreement on Trade-Related Aspects of Intellectual Property Rights (TRIPS), intellectual property (IPs) and infrastructure policy
Economic	Circular economy, Globalization, Global Economy, Global markets, Economic growth, Economic shocks, Economic support, Agricultural Economics, Hydro-economic models, Human capital, Physical capital, Intellectual Capital, Knowledge, Investments, Financing mechanisms, Interest rates, Inflation rate, Poverty, Rural poverty, Household income and revenue,, Gross revenue, Consumption pattern, Consumer preferences, Market requirements, Market trends, Exchange rates, Import/export trends, Arab markets, Price Volatility, Prices of crops, Oil price trends, Food, biofuel and fuel prices, Commodities, and Species, Trade flow and/or interruptions, Food Labor, Commercial demand for water, Recovering costs, Unemployment, Decent Work, Infrastructure, Industrial production, Agricultural production, crop production, Products, and Wealth creation
Social	Arab Communities, Arabsphere's rural communities, Regional Communities, International community, Education, Awareness, Cultural aspects, Poverty, Career attitudes, Work, Health, Family, Inequality, Regulatory frameworks, Institutional frameworks, Digital media, Communications, Information technologies, Peri-Urbanization, Law, Criminality, Punishment, Well-Being, and well-being
Technological	Innovation, Research and development (R&D), Technology development, Information technology, Digital technologies, Biomimicry, Biotechnology, Nanotechnology, Nanomaterials, Automation, Advanced production systems, Emerging technologies, and Novel technologies

In the context of Arabsphere's Water, the main Drivers categories in the future included Demographics, Ecological, Environmental, Political, Economic, Social, and Technological components. Key Factors of each category have been presented in Table 4. From a regional perspective, the “DEEPEST” road map shed light on both primary Water Resources (Rivers water, groundwater, Rains & Floods, Desalination, Reuse of wastewater, and Non-Traditional Water Resources) and Water Users and consumers (Drinking, Industry, Agricultural and Evaporation). By identifying macro-future factors, the road could be paved to procedure toward the HSP.

From water resources engineering perspective, it is not suitable to omit or summarize the factors in Table 4. When ignoring some drivers that formulate water resources needs, could cause a serious and negative impact on the ability to provide vital water resources for all potential consumers. The macro-future factors presented in Table 4 could be considered the most important drivers which affect the sustainability of the water future, and in turn, achieve sustainable development goals (SDGs) in the Arabsphere.

Three Horizons process is used to foresee Water Vision in the Arabsphere. The time intervals for first horizon H1, Second Horizon H2 and Third Horizon H3 are Less than 5 Years, within 5–10 Years and within 15–25 Years, respectively. The Theme for H1 is Water conservation WC, H2 is Circular Water CW, and H3 is Emerging Water Technologies EWTs. Examples for WC, CW, and EWTs are given in Table 5. Table 5 displays examples of possible water solutions corresponding to each horizon. The Theme for H1 is Water conservation (WC), H2 is Circular Water (CW), and H3 is Emerging Water Technologies (EWTs). Examples for WC, CW, and EWTs are given in Table 5.

**Table 5:** Water vision based on three horizons in the Arabsphere.

Three horizons	3H	Time interval	Themes	Acronym	Examples
First horizon	H1	Less than 5 years	Water conservation	WC	Water awareness and education, water metering, water saving, household water conservation, responsible water consumption, community participation, adaptive water management, gender mainstreaming, proactive water policies, transboundary cooperation strength, and Optimization water use (Xeriscaping, Hydrozoning, drip irrigation), Minimization of water waste (water-saving home devices, water evaporation, preventive maintenance of water system, water pinch analysis, Agricultural drainage water, rainwater harvesting, desalinated water, and fog harvesting
Second horizon	H2	Within 5–10 years	Circular water	CW	Physical-based, Chemical-based, Biological-based, Membrane-based, Equilibrium-based, Advanced chemical-based, Nature-based and Engineering-based Solutions. Examples include Greywater reuse, Reclaimed water, Seawater desalination, Integrated water systems (Ex. rainwater and greywater), municipal used water, and Urine-diversion toilet (UDT)
Third horizon	H3	Within 15–25 years	Emerging water technology	EWT	With respect to the Emerging Water Technologies which will be focused in the third Horizon (H3), the scope of nonconventional water resources is extremely wide, starting from deep (geologic) water cycle, deep onshore groundwater, deep offshore groundwater, reaching to upper atmosphere Emerging Water Technologies include transboundary offshore aquifers, ballast water, rain enhancement, cloud seeding, Antarctic and Arctic icebergs Harvesting (towing icebergs to the Arabsphere), Atmospheric water (AW) harvesting, Biomimetic water harvesting (WH), Bioinspired water harvesting, Bioinspired water desalination, Weather modification/control, water transportation (from other basins), Unified Super Smart Water Grid (USSWG) (a proposed water network connecting Africa, Asia and Europe continents and their countries)

Water Vision has been presented based on Three Horizons for the Arabsphere in Table 5. The main idea is to utilize the Three Horizons framework to classify freshwater resource availability as a function of cost, effort, and technical complexity, as defined in the WRs discipline. From the WRs discipline perspective, the items in the Water conservation (WC) category are “Safe” solutions which could be considered negative actions. The main idea is to save water. On the other side, the items in the Circular Water (CW) category are “Risky” solutions that require positive actions. The main idea is to treat water. Also, the items in the Emerging Water Technologies (EWTs) category are “Truly Innovative Ideas” which require positive and very complicated actions. The main idea is to harvest any drop of water. Another important issue, the Water conservation (WC) category deals with water resources acceptable for all water resources customers (drinking, hygiene, irrigation, agriculture, and industry), but in the Circular Water (CW) category different water quality parameters should be determined, and according to the actual water quality and the potential customer (drinking, hygiene, irrigation, agriculture, and industry), the cost, technology, and other significant factors could be assessed.

Designed Characteristics of Water should be identified [*Physical*: Turbidity, Color, Taste, Odor, Temperature, and Specific Conductivity; *Chemical*: Total Solids, Suspended Solids, Alkalinity, pH value, Hardness, Chlorides, Nitrogen, Phosphorus, Sulphur, Metal, chemical substances, Dissolved gases, Oil and Grease, Dissolved Oxygen (DO), Biological Oxygen Demand (BOD), Chemical Oxygen Demand (COD), Total Organic Carbon, and Adsorbable Organic Halides; *Biological*: Parasitic organisms (Bacteria, Protozoa, Algae, Viruses, Worms and Fungi)].

In general, industrial water could be classified into three grades according to comprehensive water-quality parameters: pH, BOD, COD, Total nitrogen, Total phosphorus, Total suspended solids (TSS), Total dissolved solids (TDS), Conductivity, Alkalinity as CaCO_3_, Calcium hardness as CaCO_3_, Chlorides, Sulfates, Iron (Fe), Silica, Dissolved oxygen and Fecal coliforms. In industrial sector, the technologies most commonly used include Physical, Chemical, Biological, Membrane, Equilibrium, Advanced chemical, Nature-based and Miscellaneous-based treatment. A comprehensive summary about Circular water based on 5Rs approach for WRs [reduce (losses), reuse (no treatment), recycle (treated), restore (return) and recover (other resources from used water)] has been presented^32^. Circular water technologies have been summarized in Table 6.

**Table 6:** Circular water solutions.

Solutions	Processes
Physical-based	Phase separators (water and oil mixtures separator), Sand filtration, Sand straining, Sand flocculation, Sand sedimentation, Sand surface capture, and Active carbon treatments
Chemical-based	Chemical precipitation, neutralization and Coagulation, and activated carbon adsorption (granular or powdered)
Biological-based	Biological processes, Aerobic biological assimilation, anaerobic biological treatment, and Conventional (classical) activated sludge (CAS) process
Membrane-based	Membrane filtration, separation processes, Reverse osmosis (RO), Microfiltration (MF), Ultrafiltration (UF), Ion exchange (IX) (Polishing), Nanofiltration (NF), Continuous electrode ionization (CEDI), and Organic scavenging
Equilibrium-based	Evaporation, thermal separation, incineration, and crystallization
Advanced chemical-based	Chemical oxidation process, Ultra-violet (UV) based, UV irradiation, Ultra-violet light, Photolysis, Mercury vapor lamps, Ozone-based applications, Ozonolysis, Electrophilic mechanism, and Chlorine dioxide
Nature-based	Green roofs, soil moisture retention, natural wetlands, constructed wetlands, floodplain restoration, Phytodepuration systems, groundwater recharge, and riparian buffer
Engineering-based	Zero liquid discharge process, Dissolved air flotation (DAF), Sequencing Batch Reactors (SBR), Intermittent Cycle Extended Aeration System (ICEAS), Tilted plate interceptor (TPI), Corrugated plate interceptor (CPI) (Cross Flow or Pressurized), Agitated Thin Film Dryer (ATFD), Multiple-effect Evaporator (MEE), Hydrogel solar evaporator, and Waste stabilization pond

### Emerging water technology matrix

The building of the “Emerging water technology matrix” necessitates exploring all possible and probable domains that could be significant water future opportunities. Emerging Water Technology Matrix could be constructed when focusing on the specific water resources in HSP. The grading system has four components; Excellent (E), Good (G), Pass (P), and Fail (F). The Excellent (E), Good (G), Pass (P), and Fail (F) represent Plenty, Sufficient, Vital and Deficient cases of water resources, respectively. Emerging Water Technology Matrix is shown in Table 7.

**Table 7:** Emerging water technology matrix in the Arabsphere.

Themes	First horizon	Second horizon	Third horizon
Water conservation WC	Good (G)	Excellent (E)	Excellent (E)
Circular water CW	Pass (P)	Good (G)	Excellent (E)
Emerging water technology EWT	Fail (F)	Pass (P)	Excellent (E)

## Discussion

“Futurology” has been classified as one of the Social Sciences disciplines and focused on the future of people. Now, holistic “Futures studies” are expanding to include environmental trends, S&T, and technological advancement. HS is devoted to researching water signals and their future impacts. Many new research techniques and instruments are being could be utilized in Foresight discipline. Promising examples included Big Data Analysis, Bibliometrics, Semantic Analysis, Data mining, Text mining, Technology mining, Scientometrics, and visualization (displays of the large data). Among the most significant are computer science, social science computing (SSC), computational social science (CSS), artificial intelligence (AI), business intelligence, natural language processing, machine learning, cloud computing, and application programming interfaces (APIs). Computationally, Big data sets could be analyzed to reveal trends, and patterns, relating to human consumption, behavior, production, and interactions. As an example, A future orientation index is proposed to assess the correlation between a country’s Gross domestic product (GDP) and the tendency of seeking information about the future among Internet users^33^.

### Water vision

Water Vision is a process that facilitates societies, civilizations, and countries in achieving SDGs and could be considered the backbone of an integrated water strategic framework for action. The Water vision should be a result of a huge amount of knowledge, extensive experience, long-term practice, Water wisdom, and foresight skills. Arabsphere's Water Vision for 2050 is tailored to lead to a sustainable water future, mitigate the disastrous threats, enhance coordination, cope with the future growth constraints, reduce poverty, motivate innovative water solutions, optimize value for all consumers, and stimulate social well-being and life decent. The Water Vision is devoted to the Arabsphere, water-scarce countries, countries, societies, and civilizations facing serious water challenges. From an economic perspective, Water resources could be used as a final product for vital activities such as Water, sanitation, and hygiene (WASH), or as raw and input material for cooking, irrigation, agriculture, and many industries.

The world is far from achieving SDG 6^34^. Based on the country data extracted from^34^, the 2021 status and trend of SDG 6 in the Arabsphere have been shown in Tables 8 and 9, respectively. In fact, these data show a clear picture of the existing water status which is mandatory for foreseeing Arabsphere's Water Vision. The latest data depend on collection cycles. The trend signifies a ( +) positive, ( =) no change, and (−) negative changes with regard to a specific global target. In both Tables 8 and 9, columns I, II, III, IV, V, VI, VII, VIII, IX, X, XI, and XII represent drinking water, sanitation, hygiene, domestic wastewater, industrial wastewater, water quality, water stress, integrated water management, ecosystems, transboundary water cooperation, international water cooperation, and water participation, respectively. For both Tables 8 and 9, the following Legend could be applied.

**Table 8:** Current SDG 6 status in Arabsphere.

		I	II	III	IV	V	VI	VII	VIII	IX	X	XI	XII
1	Algeria	72	18	85	76			138	54	58	11	7.5	
2	Bahrain	99	91		96	100		134	39		100	n/a	
3	Comoros							0.83	20	n/a	100	9.4	0
4	Djibouti		37		11			6.3			100	43	
5	Egypt		67	90	46			117	42		18	350	
6	Iraq	60	43	97	37			47	38	11	20	91	
7	Jordan	86	82		82		100	100	64	23	35	301	0
8	Kuwait	100	100		85			3851	94		60	n/a	
9	Lebanon	48	16				50	59	25		25	121	0
10	Libya		22			17		817	60	98	10	1.8	
11	Mauritania							13	47		10	88	2
12	Morocco	80	39		36		79	51	71	0	30	230	5
13	Oman	91		97				117	79		20	n/a	0
14	Qatar	96	97		100			431	81	0	67	n/a	
15	Saudi Arabia		59		80			993	57		14	n/a	
16	Somalia		32	25				25	22	0	25	16	
17	State of Palestine	80	67	92	48			63				114	
18	Sudan			13			86	119	34		19	62	0
19	Syrian Arab Republic			83				124	56		33	26	4
20	Tunisia	79	81	84	60		85	96	60	80	21	110	0
21	United Arab Emirates		99		96		40	1667	79	0	29	n/a	
22	Yemen		19		34			170	36		22	71	
	World	74	54	71	56		60	18	54	58	21	8846	1.2

An empty cell, there is no reported data; n/a, not applicable.

**Table 9:**
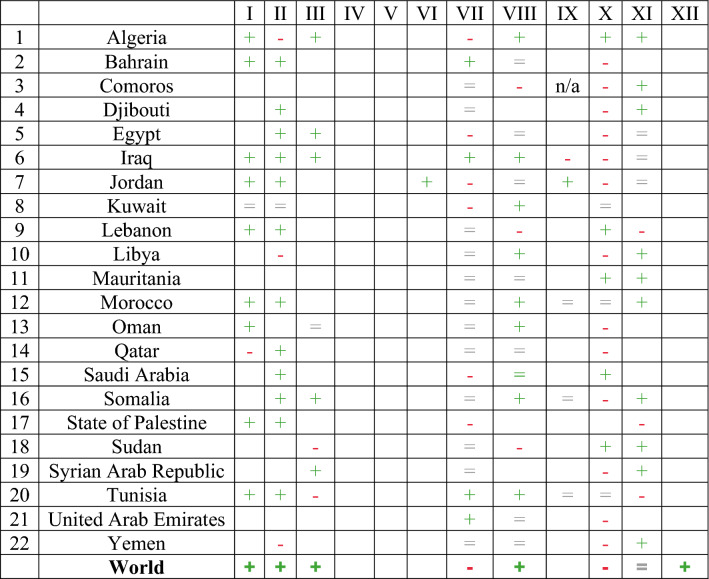
Current SDG 6 trend in Arabsphere.

(+), ; (−), ; (=), ; an empty cell, there is no reported data; n/a, not applicable.

### Water drivers of future changes

Significant drivers in Arabsphere are water vision, sustainable and climate-resistant water management, irrigation, agriculture and food (plant, animal, hunger, food, and agricultural reclamation projects), herbicide, chemical contaminants, pesticides, veterinary drug residues, infectious diseases in fish and animals, food traceability, food authenticity, anti-biotic residues, illegal and unregulated fishing, eradication of fish species, taking areas out of food production, irrigation, water quality challenges, bioinformatics, sustainable food production, food safety, food security, aquaculture products, sustainable fishing and marine resources, responsible fisheries, water policies, water use, water pollution, water reuse, drought tolerance, flood control, rainwater storage, water control methods, innovative hydraulic structures, protection of deltas, river widening, water systems, water shortages, disaster mitigation, sustainable systems, systems innovations, sustainable society, salinization, animal, plant, weed, invasive alien species, risk maps and flood risk management plans, reforestation, weather control and modification, biofuels, clean water and sanitation, water scarcity, water bill, drinking, hygiene, hand washing, used water, automatization, diagnostic technologies, integrated surveillance, innovative sensors, remote control, lab testing, risk assessment, nanomaterials, nanotechnologies, non-conventional water resources and good health.

### Circular water

Circularity (Circular economy) principle could be applied to improve water management. Used water could be the pathway to SWF and environmental sustainability. Water Circularity in the perspective of CE is the most significant emerging research topic. Water could be a main pathway to CE. Water and used water utilities can lead the pathways to SDGs, in particular SDG6 (concerning sustainable water) and SDG12 (concerning circular economy)^35^. Circular-Economy could strength sustainable water resource and recovery^36^. Combined sewer approach could improve combined sewer quality and mitigate water pollution and flooding problems, especially in crowded urban areas. Combined sewer system components are grey [flush tanks (FTs)], green [constructed wetland] and blue [receiving water body (RWB)] infrastructures^37^. Interpenetrating hydrophilic (hydratable polymeric) polymer networks have been utilized to purify water by solar energy (solar vapor generation). A durable and stable hydrogel solar evaporator could extract water from complex contaminants including heavy metal, detergents and salts components^38^.

The circular economy could enhance sustainable water management and mitigate water scarcity and nutrients shortage. Drinking water resource recovery using thermal process, chemical precipitation and reverse osmosis has been successfully examined in a pilot plant^39^. Water Circularity approach has been proposed considering economic and ecosystems and their interdependencies^40^. Used water could be a sustainable water source, nutrients, and energy resources^41^. The used water as an economic resource could support water utilities which operating sustainable water supply and sustainable sanitation systems. The World Bank initiative of concerning resource recovery devoted to transfer used water to resource has been highlighted^42^.

### Water color

Many species of water exist. Precipitation is the main freshwater source on the Earth. Pure freshwater is colorless. The color of water is a good detector for biological, chemical and physical characteristics. Impurities, suspended, particulate and dissolved materials result in discoloration. The color of water can used to diagnose the water quality status.

In case of drinking water, blue, red, green, black colors can be related to specific physical, biological and chemical drivers. Diagnosing water quality based on color spectrum analysis can make solving water problems, especially industrial used water, is a simple and straightforward process.

From irrigation perspective, green water is the precipitation consumed by non-irrigated agriculture, whereas blue water is the precipitation consumed by irrigated agriculture. Green water represents the quantity of freshwater available directly for the ecosphere (biosphere). Blue water is the quantity of freshwater available in surface water (rivers, canals, channels, etc.) and groundwater aquifers. Both “green water” and “blue water” be considered as a productive rainfall.

From domestic used water perspective, grey water is the quantity of domestic used water used at home, except urinals, bidets and toilets. Yellow water represents human urine. Brown water represents human feces without human urine. Black water represents all toilet used water, i.e. yellow water (human urine) in addition to brown water (human feces).

From industrial used water perspective, there is a spectrum of color patterns. In some conditions, all rainbow colors, Variable-colored and iridescent may be existed. The most common are white water (milky white, and whitish to greenish, blueish or brownish), green, yellow, orange, reddish-orange, red, violet, purple, yellowish to brownish, brown, dark brown, grey, and black water. The water general appearance may be quite different. Most common phenomena are Water swirls, filmy, plates, lumpy, cloudy, chalky, foamy, sudsy, scum, gelatinous, shades, dull sheen, dark, fluorescent, and rainbow sheen.

From non-conventional waters perspective, the main idea for defining the colors of domestic used water is to facilitate used water as a valuable water and economic resource. Used water as a resource may be separated into grey water, and black water (yellow water and brown water). Grey water may be reused to irrigate the gardens and green walls. Yellow water can be a source for plant nutrition as urine fertilizers. Brown water can be used to alleviate soil degradation, consequently, enhance irrigation environment.

### Non-conventional waters

In dry regions, water future necessitates application of non-conventional waters which considered base of the circular economy^43^. Non-conventional waters include desalinated water, reclaimed used water, reuse of agricultural drainage, urban or industrial used waters, fog harvesting and cloud seeding. Emerging sustainable water technologies should be environmentally compatible, socially acceptable, economically feasible and technically promising. Innovative nature-inspired water technologies include atmospheric water harvesters, biomimetic membranes, and biosaline agriculture.

Harvest icebergs could be acquired by hauling the icebergs to a suitable site in the path as an ice, where the ice converted to water, then towing this freshwater to the Arabsphere. From foresight point of view, iceberg towing to Arabsphere from Arctic or Antarctica could be a feasible solution to alleviate water stress and provide freshwater in the Arabsphere. DEEPEST holistic framework could be applied to icebergs hauling scenarios.

Advances in many scientific disciplines, engineering branches, innovation fields and technologies lead to success, achievement and superiority. Engineering fields such as Ocean engineering, Offshore engineering, Mining, Mechatronics, Power, Thermal, Mechanical, Manufacturing, Industrial, Vehicle, Materials, Electronic, Structural, Transport, Information, Safety, Reliability, Systems engineering, Supply chain engineering, Quantum engineering, applied engineering, project engineering, and Engineering management are promising in this vision. Technological advances in fields such as artificial intelligence, computer modelling and simulation, computer-aided design, automation, robotics, artificial satellites, control systems, and super powerful rig towing tug can make a difference. Financial institution, insurance companies, and investment banks could support scenarios of icebergs hauling and offer palatable economic risk-free mechanism. Greenhouse gas and carbon footprint should be mitigated to allowable international standards.

### Atmospheric water harvesting (AWH)

One of the most promising Emerging Water Technologies is Atmospheric Water Harvesting (AWH). Recently, many scholars have made significant contributions in this optimistic field. AWH is emerging technology to mitigate the global water scarcity. AWH is constructed to extract water. Arid Air Water Harvesting by using composite sorbent made of hygroscopic salt and Metal–Organic Frameworks is an emerging water technology^44^. AWH using Metal organic frameworks as adsorbents is a promising device in the light of temperature and pressure responses, and working capacity^45^. Atmospheric water could be harvested from air by nano sorbent. Multiple water harvesting cycles could be achieved for effective applications by nano-carbon shell^46^. Hydrogels are emerging materials for atmospheric water harvesting. Water could be absorbed and evaporated within hydrogels. Hydrogels have tailor-made physiochemical characteristics.^47^. Atmospheric water could be harvested by nano biopolymer hygroscopic aerogel have high-capacity water storage using lyophilization (freeze-drying/cryodesiccation) process in dry, cool, and nature sunlight severe environmental outdoor conditions^48^.

Adsorption Water Harvesting systems could be effective by applying Advanced Metal–organic frameworks^49^. Atmospheric water could be harvested by water sorption process. Polymeric sorbent, which enhance performance and productivity, and free of metal and halide has been proposed^50^. The water harvester has been provided by non-toxic and bio-degradable desiccant^51^. Hygroscopic, Inorganic porous materials and Organic sorbents have successfully been used in atmospheric water harvesting. Sorbent-based water harvesting systems have significant design properties such as absorbency, stability, host materials, quantity, regeneration, relative humidity, maximum water harvesting, cost, safety, life cycle and technique^52^.

Recently, solar‐driven AWH has been emerged as an innovative discipline. Extraction of water vapor could be effective process to purify and produce freshwater when applying solar energy^53^. AWH is developed by inspiring the biological adaptability of some plant species that could absorb moisture using hygroscopic photothermal organogel powered by solar technique^54^. AWH is developed by desiccant-based, solar-driven model to reduce energy requirement^55^. AWH in arid regions is developed by Solar-Driven Dual-Stage Device utilizing advanced performing adsorbents to maximize water production and minimize heat losses^56^.

### Humidity harvesting systems

Humidity Harvesting system utilizing a porous framework has been suggested. The system could purify moisture captured from contaminated air environment or atmospheric environment^57^. Advanced dehumidifiers could be effective devices for sustainable freshwater production, dehumidification and raising the thermal comfort. High humidity could be utilized as a freshwater resource to alleviate water scarcity.^58^. Humidity Harvesting systems depends on successful water adsorption. Emerging Water adsorbents involve porous organic polymers, metal–organic structures, hydrogen-bonded organic structures, covalent organic structures, bioinspired nanostructures, nano-porous water-absorbent gels, controlled morphologies nanomaterials, nanofibers, nanorods, and two-dimensional nanosheets materials. The physicochemical characteristics of merging Water adsorbents for water capture by dehumidification such as hydrophilicity, stability, binding enthalpy, surface areas, water uptake and tunable functionalities are extremely significant when designing such porous organic polymers materials^59^.

AW irrigation process using solar-powered for sustainable farming has been proposed. Super Moisture Absorbent Gels could harvest AW and irrigate the plants. Atmospheric water irrigation process causes the agriculture in drought and arid areas could overcome distant and/or remote water supplies^60^. AWH could be applied to enhance the performance of Green Roofs. Integrated green roof with fog harvesting (FH) and dew harvesting (DH) systems to enhance the performance of Green Roofs has been proposed^61^. AW in island regions could be harvested by air-cooled water device^62^.

Practical considerations are extremely important. The water source could be precipitation, fog, dew, or humidity. The innovative system could be manufactured, fabricated, installed, built, constructed or implemented in the site. Of course, the main concern is the WH capacity of technology, i.e. the total volume of water could be supplied per day. DEEPEST holistic framework could be applied to investigate challenges and sustainability aspects. Water sources, water treatment if any, community needs, target users, market price, design specifications, technical support, life cycle, safety, performance, spare parts, materials, energy requirement, hazards, quality systems and environmental conditions are significant elements.

### Shared waters

The majority of the Arabsphere’s fresh water originates outside their political borders. Shared waters are serious to Arabsphere and must be cosidered as a tool for building cooperation and peace. Equitable allocation of WRs and exchange benefits through dialogue and negotiations are the key for active regional cooperation among countries in the Arabsphere and their neighbors. The Arabsphere needs to examine how to enhance cooperation and integration. The Arabsphere should develop innovative schemes for conflict resolution. Water balance should be explored at shared basins including both green and blue waters. Utilization of all waters including rivers, surface water, groundwater, blue-water, and green water, should be equitable and reasonable. IWRM, WUE and Water Nexuses should be monitored and supported.

### Water's horizons

The category of Water conservation techniques is the cheapest, easiest, and technologically simplest, so it is most suitable to be applied in the First Horizon. Comparably, the category of Circular Water techniques is more costly, harder, and technologically more complex, so it could be suitable to be applied in the Second Horizon. Similarly, the category of Emerging Water Technologies is the costliest, hardest, and most technologically complex, then it is hoped to be suitable to be applied in the Third Horizon. So, the decision-maker in the WRs discipline could emphasize technologies related to Water conservation in First Horizon (less than 5 years), technologies related to Circular Water in Second Horizon (within 5–10 years), and technologies related to Emerging Water Technology in Third Horizon (within 15–25 years). The items in the Emerging Water Technologies (EWTs) category are “Truly Innovative Ideas” which require positive and very complicated actions, the main idea is to harvest any drop of water.

## Conclusions

Availability of water resources is the most complicated challenge facing sustainable development in the Arabsphere. “Foresight” could be considered as the ability to foresee the future wisely. The application of “Futurology” principles and methodologies in the WRs discipline, which mainly belongs to civil and environmental engineering discipline leads to an interdisciplinary study, which in turn necessitates paving the road, and bridging the knowledge gap between Social Sciences and engineering disciplines, and practicing some sort of common language. The present study focused on United Nations SDGs—in particular, SDG 6 and SDG12—to achieve the strategic visions of the Arabsphere. From water resources engineering perspective, there are many opportunities to provide water resources. It is a common-sense practice to prioritize criteria for choosing the most suitable alternative, in terms of all Drivers, and in particular, Demographics, Ecological, Environmental, Political, Economic, Social, and Technological. From the WRs discipline perspective, there is some sort of analogy between Water conservation (WC), Circular Water (CW), and Emerging Water Technologies (EWTs) from one side and “Safe” solutions, “Risky” ideas, and “Truly Innovative Ideas”, from another side. Although the individual brainstorming started with “Safe” solutions, it continued with “Risky” ideas, hoping to achieve “Truly Innovative Ideas”. The wild ideas have guided the survey of future water developments and suggested more water resource opportunities and explored innovative water resource solutions. Some examples of wild ideas have been deep offshore groundwater, Unified Super Smart Water Grid (USSWG), and Antarctic and Arctic icebergs Harvesting. Now, some emerging water technologies which successful at the laboratory scale could be insignificant as a water resource for the Arabsphere water balance. In the future, those emerging water technologies could perfectly support the Arabsphere's Water Vision. Actually, achieving the SDGs necessitates successful integration between foresight methodologies and the decision-making process. Although the HSP is not a magic wand to create precious freshwater resources, Hopefully, HSP is a base for a wise Water Vision in the Arabsphere. The Proactive vision of this study is to promote Foresight discipline in academia, technology, industry, and business. The outcomes of the application of the HSP to foresight emerging issues in Arabsphere's Water Vision could be a pioneering example for other areas of Sustainable Development and other United Nations SDGs. The successor examples could emphasize achieving no-regrets futures, avoiding extreme weather and disruptions, and mitigating Climate Change. From a future-relevant perspective, similar assessments could be implemented for other SDGs. Food (SDG 2), energy (SDG 7), and cities (SDG 11) could be obvious and straightforward applications. Similar approaches for resources, especially, climate, water, food, energy, and land nexus could be applicably fruited to enable sustainable pathways and the furtherance of the SDGs for the future.

## Data Availability

The datasets used and/or analyzed are available from the author on reasonable request.

## Abbreviations

3H: Three horizons
3HP: Three horizons process
5Rs: 5 R approaches for water resources WRs [reduce (losses), reuse (no treatment), recycle (treated), restore (return) and recover (other resources from used water)]
A: Agriculture
AI: Artificial intelligence
APIs: Application programming interfaces
*A*_r_: The ratio of water used by the agriculture sector over the total water uses
ASRT: Academy of Scientific Research & Technology
ATFD: Agitated thin film dryer
AW: Atmospheric water
AWH: Atmospheric water harvesting
*A*_WUE_: Index of water-use efficiency of the agriculture sector
BOD: Biological oxygen demand
CaCO_3_: Calcium carbonate
CAS: Conventional (classical) activated sludge process.
CC: Climate change
CE: Circular economy
CEDI: Continuous electrode ionization
COD: Chemical oxygen demand
CoV: Coronaviruses
COVID-19: Coronavirus pandemic
CPI: Corrugated plate interceptor
CSS: Computational social science
CW: Circular water
DAF: Dissolved air flotation
DEEPEST: Demographics, ecological, environmental, political, economic, social, and technological
DESTEP: Demographic, economic, socio-cultural, technological, ecological and political
DH: Dew harvesting
DO: Dissolved oxygen
E: Excellent
EC: European Commission
EIs: Emerging issues
EKB: Egyptian Knowledge Bank
ES: Economic sector
ES_r_: The ratio of water utilized by an economic sector (ES) over the total water uses
ES_WUE_: Index of water-use efficiency for an economic sector
EU: European Union
EWT: Emerging water technology
EWTM: Emerging water technology matrix
EWTs: Emerging water technologies
F: Fail
FAO: Food and Agriculture Organization of the United Nations
Fe: Iron
FH: Fog harvesting
FP: Foresight process
FTs: Flush tanks
G: Good
GCPSE: Global Centre for Public Service Excellence
GDP: Gross domestic product
H1: 1st horizon, the first horizon “current if remains in future”
H2: 2nd horizon, the second horizon “mid-term”
H3: 3rd horizon, the third horizon “long-term”
HHS: Humidity harvesting systems
HS: Horizon scanning
HSP: Horizon scanning process
I: Industry
ICEAS: Intermittent cycle extended aeration system
IPs: Intellectual property
*I*_r_: The ratio of water used by the Industry sector over the total water uses
IRM: Institute of Risk Management
IWA: International Water Association
IWRM: Integrated Water Resources Management
*I*_WUE_: Index of water-use efficiency of the industry sector
IX: Ion exchange (polishing)
LDCs: Least developed countries
LST: Land surface temperature
MEE: Multiple-effect evaporator
MF: Microfiltration
MFF: Macro-future factors
NF: Nanofiltration
P: Pass
PEST: Political, economic, socio-cultural and technological
PESTE: Political, economic, social, technological, and environmental
PESTEL: Political, economic, socio-cultural, technological, legal and environmental
PESTLE: Political, economic, social, technological, legal and environmental
PESTLEE: Political, economic, socio-cultural, technological, legal, ethics, and ecological
pH value: Potential (power) of hydrogen (in chemistry), a scale to specify the acidity or basicity of a chemical solution.
PMESII-PT: Political, military, economic, social, infrastructure, information, physical environment, and time (US army)
R&D: Research and development
RDI: Research, development and. innovation
RO: Reverse osmosis
RWB: Receiving water body
S: Services
S&T: Science and technology
SBR: Sequencing batch reactors
SD: Sustainable development
SDG 6: Sustainable development goal on water and sanitation
SDG12: Sustainable development goal 12 (Goal 12. Ensure sustainable consumption and production patterns)
SDG6: Sustainable development goal 6 (Goal 6. Ensure availability and sustainable management of water and sanitation for all)
SDGs: Sustainable development goals
SLEPT: Socio-cultural, legal, economic, political and technological
SPELIT: Socio-cultural, political, economic, legal, intercultural, and technological
*S*_r_: The ratio of water used by the Services sector over the total water uses
SSC: Social science computing
STDF: Science, Technology & Innovation Funding Authority
STEEP: Social, technological, economic, environmental and political
STEEPLE: Socio-cultural, technological, economic, ethics, political, legal, and ecological
STEEPLED: Socio-cultural, technological, economic, ethics, political, legal, ecological and demographic
STEER: Sociocultural, technological, economic, ecological, and regulatory
STEPE: Socio-cultural, technological, economic, political and ecological
STI: Science, technology, and innovation
SW: Shared waters
SWF: Sustainable water future
*S*_WUE_: Index of water-use efficiency of the services sector
TDS: Total dissolved solids
TELOS: Technical, economic, legal, operational, and scheduling
TIIs: Truly innovative ideas
TPI: Tilted plate interceptor
TRIPS: The Agreement on Trade-Related Aspects of Intellectual Property Rights
TSS: Total suspended solids
UDT: Urine-diversion toilet
UF: Ultrafiltration
UK: United Kingdom
UN: United Nations
UNEP: United Nations Environment Programme
UNESCO: United Nations Educational, Scientific and Cultural Organization
UNESCWA: United Nations Economic and Social Commission for Western Asia
USA: United States of America
USD/m^3^: United States dollar per cubic meter (of water)
USSWG: Unified super smart water grid
UV: Ultra-violet
WASH: Water, sanitation, and hygiene
WBCSD: World Business Council for Sustainable Development
WC: Water conservation
WH: Water harvesting
WRM: Water resources management
WRs: Water resources
WUE: Water-use efficiency
WWAP: World Water Assessment Programme
